# *Hes7* 3′UTR is required for somite segmentation function

**DOI:** 10.1038/srep06462

**Published:** 2014-09-24

**Authors:** Takeshi Fujimuro, Takaaki Matsui, Yasuhide Nitanda, Tatsuro Matta, Yuichi Sakumura, Michiko Saito, Kenji Kohno, Yasukazu Nakahata, Yasumasa Bessho

**Affiliations:** 1 Laboratory of Gene Regulation Research, Graduate School of Biological Sciences, Nara Institute of Science and Technology, Ikoma, Nara 630-0192, Japan; 2 Biological Systems Design Laboratory, School of Information Science and Technology, Aichi Prefectural University, Nagakute, Aichi 480-1198, Japan; 3 Laboratory of Neuronal Cell Morphogenesis, Graduate School of Biological Sciences, Nara Institute of Science and Technology, Ikoma, Nara 630-0192, Japan; 4 Laboratory of Molecular and Cell Genetics, Graduate School of Biological Sciences, Nara Institute of Science and Technology, Ikoma, Nara 630-0192, Japan

## Abstract

A set of genes in the posterior end of developing mouse embryos shows oscillatory expression, thereby regulating periodic somite segmentation. Although the mechanism for generating oscillation has extensively been clarified, what regulates the oscillation period is still unclear. We attempted to elongate the oscillation period by increasing the time to transcribe *Hes7* in this research. We generated knock-in mice, in which a large intron was inserted into *Hes7* 3′UTR. The exogenous intron was unexpectedly not properly spliced out and the transcripts were prematurely terminated. Consequently, *Hes7* mRNA lost its 3′UTR, thereby reducing the amount of Hes7 protein. Oscillation was damped in the knock-in embryos and periodic somite segmentation does not occur properly. Thus, we demonstrated that *Hes7* 3′UTR is essential to accumulate adequate amounts of Hes7 protein for the somite segmentation clock that orchestrates periodic somite formation.

Somitogenesis is the most prominent cyclic event in vertebrate development. Somites are sequentially formed in the anterior-posterior direction. A pair of somites buds off from the anterior part of the presomitic mesoderm (PSM), i.e., the unsegmented mesenchymal tissue in the posterior embryo of vertebrates, in a rhythmic fashion. Due to this temporal periodicity, the array of somites is formed as a spatial periodic pattern. Somites give rise to vertebrae, ribs, skeletal muscles, and skin, and thus, somites are the earliest segmental units of the vertebrate body^1^,^2^. The period of somite formation is characteristic of the species, e.g., 2 h in mice, 90 min in chicks, and 20–30 min in zebrafish^3^. Because the timing for the beginning and end of somite formation is strictly determined during development, the number of somites and the number of resulting vertebrae may be dependent on the period of somitogenesis.

The periodicity of somitogenesis is instructed by the synchronous oscillation of gene expression in PSM, which is termed the somite segmentation clock^1^. The expression of a set of genes oscillates in a 2-h cycle in mouse PSM, where expression demonstrates cyclic wave-like propagation from the posterior end of the embryo to the anterior PSM. A pair of somites is generated during each cycles^3^. The oscillating genes in mouse include components of Notch signaling, fibroblast growth factor (FGF) signaling, and Wnt signaling^4^. One of these genes is an effector gene of Notch signaling, *Hes7*, which encodes a basic helix-loop-helix type transcription factor^5^. Hes7, as a transcriptional repressor, binds to its own promoter, thereby inhibiting its own transcription^6^,^7^. Thus, Hes7 forms a negative feedback loop, which is the major mechanism for oscillatory gene expression. Hes7 also inhibits the transcription of several target genes; therefore, the expression of these genes oscillates in a synchronized manner^7^. These target genes of Hes7 include *lunatic fringe* (*Lfng*), which encodes a glycosyl-transferase that modulates the activity of Notch signaling^8^. The activity of Notch signaling also oscillates in the PSM due to the oscillation of Lfng, and this dynamic Notch activity contributes to the mechanism for somite segmentation^9^.

Mathematical models have been proposed based on this mechanism for the transcriptional regulation that generates oscillatory gene expression in the somite segmentation clock^10^,^11^,^12^,^13^. Of these, a mathematical model that directed the negative feedback loop of a transcription factor with its time delay successfully reproduced oscillatory gene expression^12^,^13^. The predicted period of oscillation in this model mainly depended on the time delay of the negative feedback loop. The time delay included the time taken to transcribe and process mRNAs, synthesize and modulate proteins, and traffick mRNAs and proteins. One acceptable way to test and verify the model, in which the feedback loop of Hes7 plays a central role, is to manipulate parameters in the model and to detect its resulting phenotype in somitogenesis. For instance, increasing the time for mRNA transcription should increase the time delay, thereby extending the period of gene oscillation and somite segmentation in the model.

We tried to increase the period of gene oscillation and somite segmentation in mouse somitogenesis in this research by increasing the time taken to transcribe *Hes7*. We inserted a large intron derived from human *dystrophin* into the 3′UTR region of mouse *Hes7* in ES cells, and generated knock-in mice. Previous researches have attempted to increase the time delay in the somite segmentation clock by using similar strategies. Stauber et al. inserted large intron sequences derived from human *dystrophin* into the third intron of mouse *Hes7*^14^, and Hanisch et al. introduced a *her1* transgene that they inserted a large sequence into the second intron into a *her1*/*her7* mutant zebrafish^15^. The large introns were unexpectedly not correctly spliced out in both studies, and both groups failed to increase the time delay in the somite segmentation clock. The exogenous intron was coincidentally not properly spliced out in this study, and thus we also failed to increase the time taken to transcribe *Hes7*. The transcripts of the knock-in *Hes7* allele were prematurely terminated within the intron sequence in the mouse PSM cells. Consequently, *Hes7* mRNA lost its 3′UTR. Oscillatory gene expression in PSM was lost in the embryos of the knock-in mice and no adequate periodic somite segmentation occurred. Accordingly, the metameric pattern of somites was severely affected, thereby severely disrupting the axial skeletons. This phenotype is similar to that in *Hes7* null mutants. In addition, the amount of Hes7 protein was severely reduced in the PSM of the knock-in mice. Thus, *Hes7* 3′UTR was essential to generate sufficient amounts of Hes7 protein. Altogether, our results demonstrated that *Hes7* 3′UTR is required for the somite segmentation function.

## Results

### Generation of Knock-in mice

**Figure 1:**
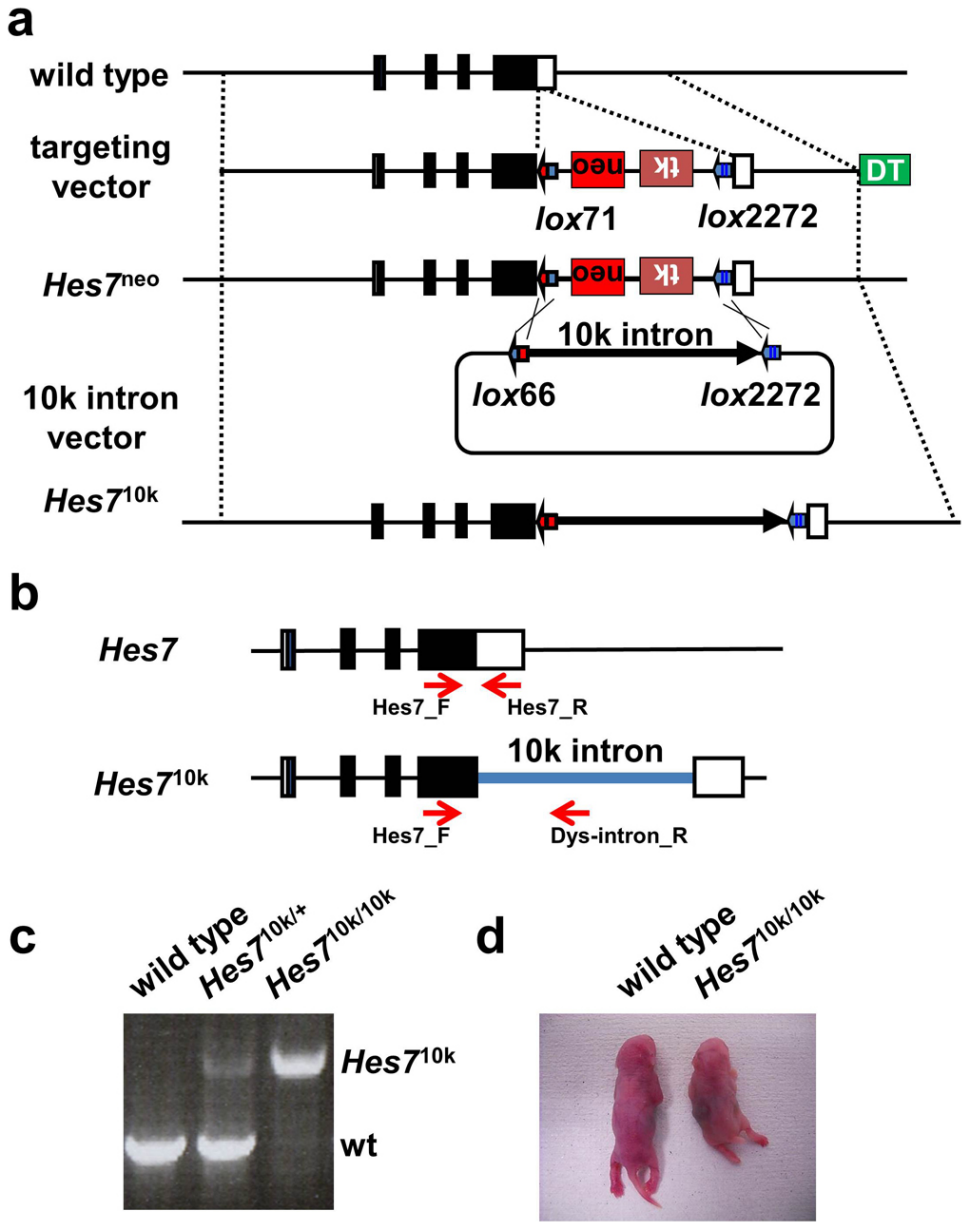
Generation of *Hes7*^10k/10k^ mice. (a) Targeting strategy. The top line indicates the structure of the wild-type *Hes7* gene and the second line indicates the structure of the targeting vector. *lox*71, PGK-*neo*, PGK-*TK*, and *lox*2272 were inserted into the *Hes7* 3′UTR region. PGK-*neo* and PGK-*TK* were placed in reverse orientation. The third line indicates the resulting knock-in allele, *Hes7*^neo^. The fourth line indicates the vector that includes human *dystrophin* intron with *lox*66 and lox*2272*. *lox*71 and *lox*2722 in *Hes7*^neo^ were rearranged with *lox*66 and lox*2272*, respectively, thereby creating the knock-in allele, *Hes7*^10k^, which is on the bottom line. The *diphtheria toxin* gene (DT) was used for negative selection. (b,c) Genotype analysis by PCR. The positions of PCR primers are indicated by the red arrows. The combination of the primers Hes7_F and Hes7_R detect a 540-bp fragment (wt) in the wild-type. The combination of the Hes7_F and Dys-intron_R detect a 1570-bp fragment (*Hes7*^10k^) in the *Hes7*^10k^ allele. (d) A wild-type neonate and a *Hes7*^10k/10k^ neonate. The *Hes7*^10k/10k^ neonate has a short trunk and a short tail.

Hes7 protein inhibits its own transcription to form a negative feedback loop, which is supposed to be the core mechanism for gene oscillation^7^. Previous research using mathematical modeling has proposed that the time delay from *Hes7* transcription to the accumulation of Hes7 protein is the critical factor in determining the oscillation period^13^. We tried to lengthen the time delay by increasing the size of the *Hes7* gene to verify the prediction, and observed whether the oscillation period was extended. We inserted 5, 10, and 20 kb exogenous intron sequences derived from human *dystrophin* into embryonic stem (ES) cells at the 3′UTR of the *Hes7* gene by homologous recombination (Fig. 1a: see Methods). The recombinants were confirmed by Southern blotting and polymerase chain reaction (PCR) analyses (Fig. 1b,c, data not shown). The transcription elongation rate of RNA polymerase II was previously estimated by several groups, and they worked out different values from 1.1–4.8 kb/min^15^,^16^,^17^,^18^,^19^,^20^. If we had taken 1.1 kb/min as the velocity of the RNA polymerase on the *Hes7* allele on the one hand, we would have expected that 5, 10, and 20 kb exogenous intron sequences would respectively increase the time delay by 4.5, 9, and 18 min. If we had assumed it to be 4.8 kb/min on the other hand, we would have expected that 5, 10, and 20 kb exogenous intron sequences would respectively increase the time delay by 1, 2.1, and 4.2 min. We carried out a simulation according to our mathematical model to estimate the expected oscillation period with these alterations^21^ (Supplementary note). Our model predicted that the increment of time delay led to sustained oscillations, and that 2.1–9 min additional time delay brought by the 10 kb exogenous intron sequence increased the oscillation period by 4.9–20.2 min. The exogenous intron sequences were expected to be spliced out, and consequently only 89-base insertion derived from *lox*P and the human *dystrophin* sequence should remain in the 3′UTR of the mature transcripts from the mutant *Hes7* allele. The resulting Hes7 protein should be exactly the same as that of the wild type.

Mutant mice were generated from the recombinant ES cells. We found that *Hes7*^5k/5k^ mice, *Hes7*^10k/10k^ mice, and *Hes7*^20k/20k^ mice had short trunks and tails (Fig. 1d, data not shown). The short trunks and tails were unexpected phenotype because our mathematical simulation predicted that increasing the time delay would lead to the oscillation period being altered, but that sustained oscillation would still be maintained.

### Homozygous mutant mice with defects in somite formation and consequent segmental defects

**Figure 2:**
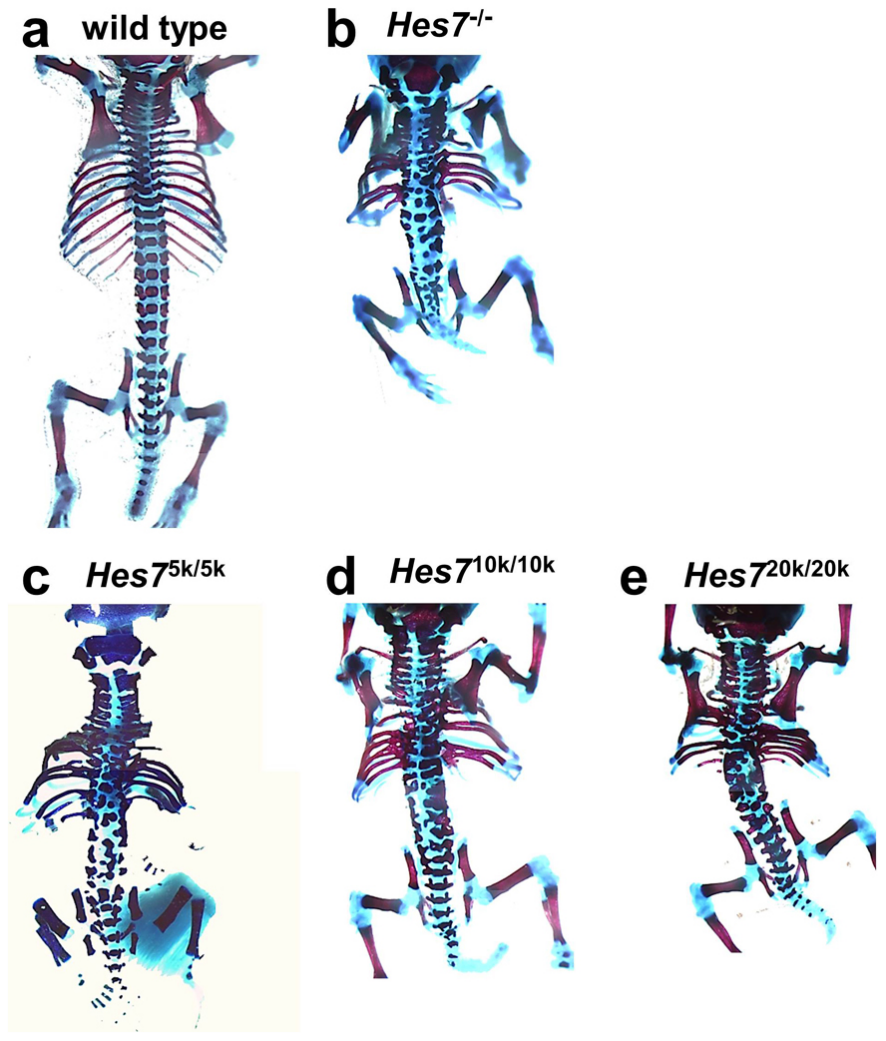
Axial-skeleton defects in *Hes7* knock-in mice. Bone (red) and cartilage (blue) staining for newborn mice of wild-type (a), *Hes7*^-/-^ (*n* = 3) (b), *Hes7*^5k/5k^ (*n* = 3) (c), *Hes7*^10k/10k^ (*n* = 3) (d), and *Hes7*^20k/20k^ (*n* = 3) (e) neonates.

Because the phenotypes of short trunks and tails resembled those of *Hes7* null mutant mice (*Hes7*^-/-^)^5^, which have anomalous axial skeletons, we examined the axial patterning in *Hes7*^5k/5k^, *Hes7*^10k/10k^, and *Hes7*^20k/20k^ neonates. The vertebrae and ribs of neonates were stained with alizarin red and alcian blue. All *Hes7*^5k/5k^, *Hes7*^10k/10k^, and *Hes7*^20k/20k^ neonates had segmentation defects in their vertebrae and ribs, and their phenotypes were similar (Fig. 2c–e). Segmental defects were observed throughout the vertebrae and ribs of all *Hes7*^5k/5k^, *Hes7*^10k/10k^, and *Hes7*^20k/20k^ neonates, which were the same as the skeletal phenotypes in *Hes7*^-/-^ neonates. However, the axial skeletons of *Hes7* null neonates shrank much more than those of *Hes7*^5k/5k^, *Hes7*^10k/10k^, and *Hes7*^20k/20k^ neonates (Fig. 2b–e). We expected that the *Hes7*^5k/5k^, *Hes7*^10k/10k^, and *Hes7*^20k/20k^ mice would have longer oscillation periods from our mathematical simulation, which would lead to fewer somites/vertebrae. In addition, because the expected oscillation period in *Hes7*^20k/20k^ mice was longer than that in *Hes7*^10k/10k^ or *Hes7*^5k/5k^ mice, the *Hes7*^20k/20k^ mice were expected to have fewer vertebrae than in *Hes7*^10k/10k^ or *Hes7*^5k/5k^ mice. However, there were no differences in the numbers of vertebrae in *Hes7*^5k/5k^, *Hes7*^10k/10k^, and *Hes7*^20k/20k^ neonates. We decided to examine *Hes7*^10k/10k^ mice in the analyses that followed because the phenotypes of all *Hes7*^5k/5k^, *Hes7*^10k/10k^, and *Hes7*^20k/20k^ neonates were similar.

**Figure 3:**
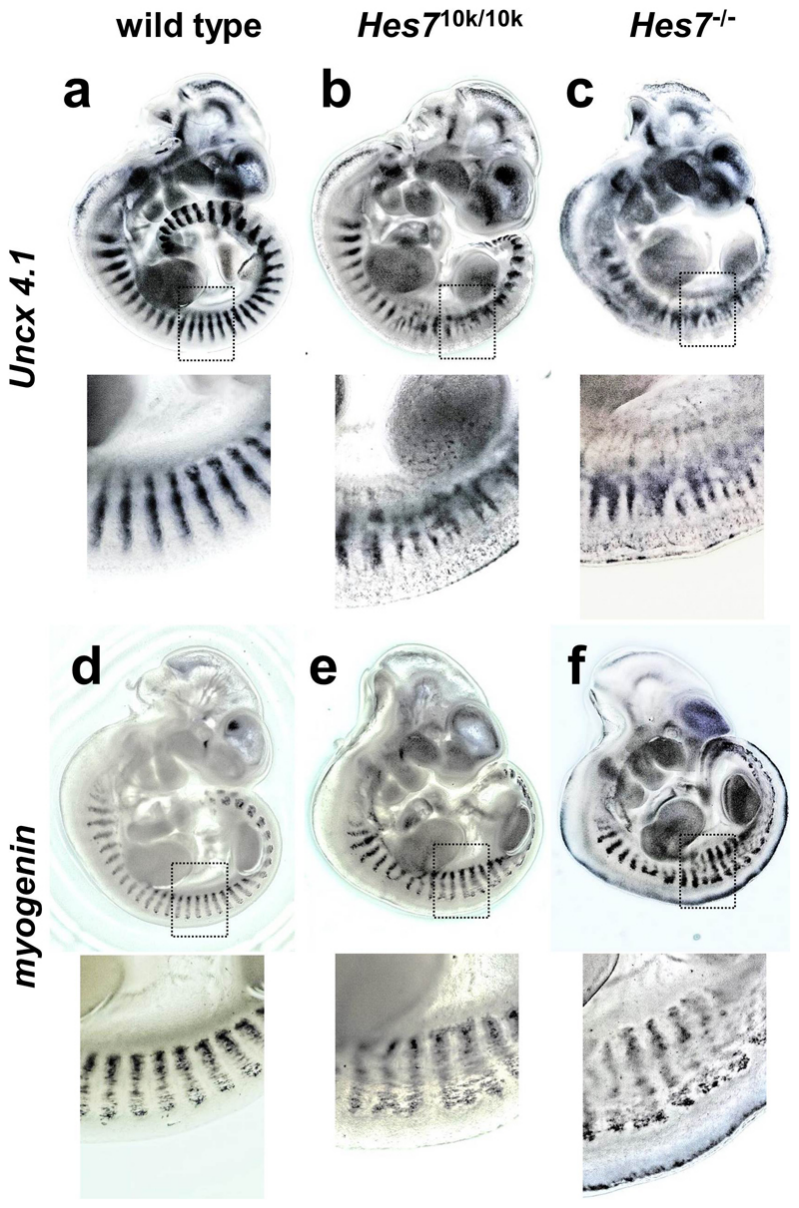
Defects in somite segmentation in *Hes7*^10k/10k^ mice. Expression patterns of *Uncx4.1* at E 10.5 in wild-type (*n* = 25) (a), *Hes7*^10k/10k^ (*n* = 15) (b), and *Hes*7^-/-^ (*n* = 6) (c) embryos. Expression patterns of *myogenin* at E 11.5 in wild-type (*n* = 13) (d), *Hes7*^10k/10k^ (*n* = 7) (e), and *Hes*7^-/-^ (*n* = 5) (f) embryos. The lower panels are enlarged views of the areas of dotted squares in each upper panel.

Because the segmental patterns of vertebrae and ribs were derived from the metameric pattern of somites in the embryonic stage, we next examined somite patterning in *Hes7*^10k/10k^ embryos. A homeobox gene, *Uncx4.1*, was exclusively expressed in the posterior half of each somite, thereby displaying an ordered ladder pattern in the wild-type embryos (Fig. 3a). The expression of *Uncx4.1* was severely disrupted in the absence of *Hes7* (Fig. 3c), as has previously been reported^5^. The signals of *Uncx4.1* were fused in *Hes7*^10k/10k^ embryos and the ladder pattern was disrupted (Fig. 3b). Another somite marker, *myogenin*, which was expressed in the myotome in each somite, also exhibited an ordered ladder pattern in wild-type embryos (Fig. 3d). However, the signals of *myogenin* were fused in some parts in *Hes7*^10k/10k^ embryos (Fig. 3e) as same as in *Hes7*^-/-^ embryos (Fig. 3f). These results indicate that somites are not sufficiently segmented in *Hes7*^10k/10k^ embryos, and this anomaly in somites probably leads to segmental defects in neonates.

### Amount of Hes7 protein is reduced in *Hes7*^10k/10k^ embryos

**Figure 4:**
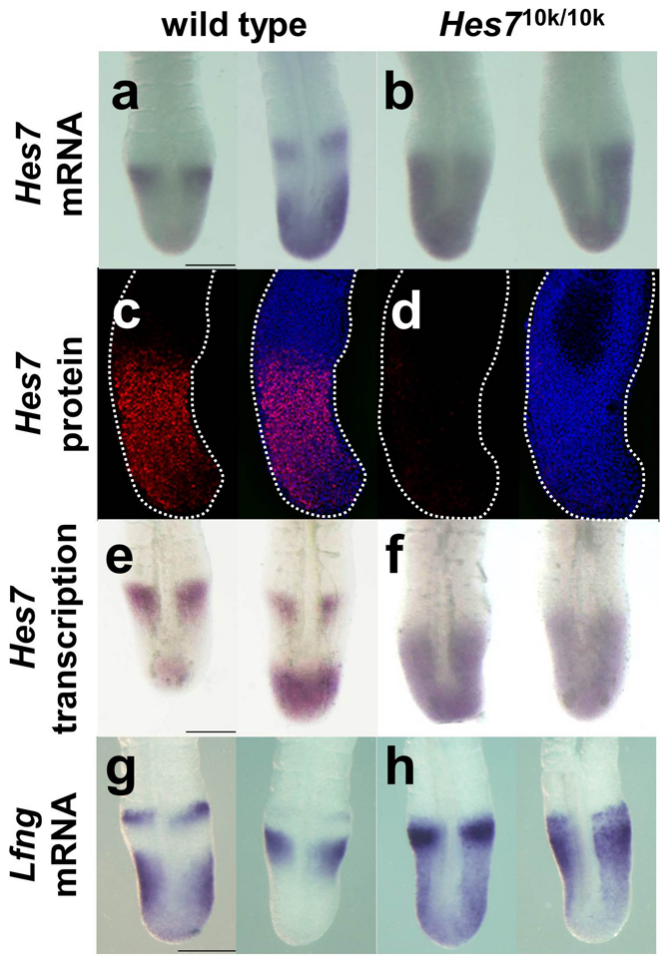
Expression patterns of *Hes7* and *Lfng*, and distribution of Hes7 protein in PSM. (a,b) Distribution of *Hes7* mRNA in PSM of wild-type (*n* = 6) (a) and *Hes7*^10k/10k^ (*n* = 3) (b) embryos at E 10.5. (c,d) Distribution of Hes7 protein in PSM of wild-type (*n* = 15) (c) and *Hes7*^10k/10k^ (*n* = 17) (d) embryos at E 10.5. Whole-mount immunostaining was carried out with anti-Hes7 antibody. Lateral views were shown, and white dotted lines represent the contours of PSM. *Left* panels show the signal for Hes7 immunostaining and *right* panels show the merged views with the nuclei stained with Hoechst33258 (e,f) Regions active in the transcription of *Hes7* detected using *Hes7* intron probe in PSM of wild-type (*n* = 5) (e) and *Hes7*^10k/10k^ (*n* = 4) (f) embryos at E 10.5. (g,h) Distribution of *Lfng* mRNA in PSM of wild-type (*n* = 12) (g) and *Hes7*^10k/10k^ (*n* = 8) (h) embryos at E 10.5. Scale bars: 100 μm.

We detected *Hes7* mRNA with a probe derived from the *Hes7* coding sequence in wild-type and *Hes7*^10k/10k^ embryos at E 10.5 to check the expression patterns of cyclic genes in *Hes7*^10k/10k^ embryos. The expression of *Hes7* revealed wave patterns in the PSM of wild-type embryo, as has previously been reported^5^ (Fig. 4a). In contrast, the expression of *Hes7* mRNA spread uniformly in PSM of *Hes7*^10k/10k^ embryos, and did not exhibit wave patterns (Fig. 4b).

**Figure 5:**
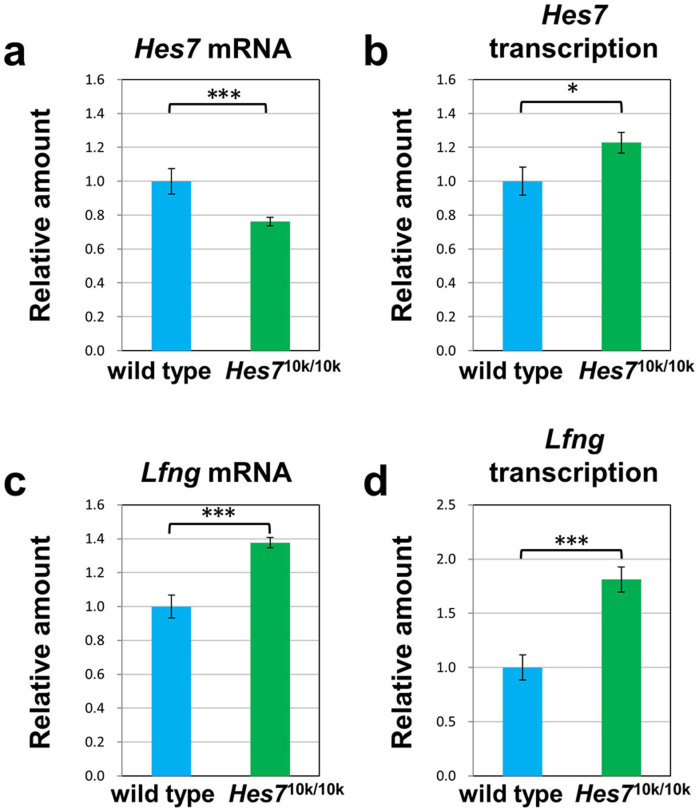
Quantitative analyses for amount of mRNA and transcriptional activity of *Hes7* and *Lfng* in PSM at E 10.5. Total RNA was extracted from the PSM of wild-type or *Hes7*^10k/10k^ embryo at E 10.5, and the amount of *Hes7* mRNA (a), nascent transcripts of *Hes7* (b), *Lfng* mRNA (c), and nascent transcripts of *Lfng* (d) in each PSM was measured by quantitative RT-PCR. The transcriptional activity of *Hes7* and *Lfng* were assessed by the number of nascent transcripts of *Hes7* and *Lfng*, respectively, that was quantified by quantitative RT-PCR detecting intron sequences. The amount of mRNA or nascent transcripts was normalized by the amount of *Glyceraldehyde 3-phosphate dehydrogenase (GAPDH)*, and the relative value to the value for wild type was represented. Each data point represents the means ± s.e.m. of 14 (wild type) and 13 (*Hes7*^10k/10k^) PSMs. (***p < 0.01, *p < 0.05; Student's t-test).

We speculated that the amount of *Hes7* mRNA and/or Hes7 protein in *Hes7*^10k/10k^ embryos would be insufficient to maintain sustained oscillation because the transcription of *Hes7* is activated through the PSM cells in *Hes7* null embryos^7^. Thus, we assessed the amount of *Hes7* mRNA and Hes7 protein derived from the *Hes7*^10k^ allele in *Hes7*^10k/10k^ embryos. We found from quantitative PCR that the amount of *Hes7* mRNA was reduced by 30% in the PSM of *Hes7*^10k/10k^ embryos in comparison with wild-type embryos (Fig. 5a). We then carried out immunohistochemistry with an anti-Hes7 antibody. The Hes7 protein in wild-type embryos revealed a wave pattern in the PSM (Fig. 4c). In contrast, we could hardly detect Hes7 protein in the PSM of *Hes7*^10k/10k^ embryos (Fig. 4d). These results suggest that the amount of Hes7 protein derived from the *Hes7*^10k^ allele was grossly reduced in PSM, and that Hes7 protein was not effectively produced in *Hes7*^10k/10k^ embryos. This is consistent with the phenotypes of *Hes7*^10k/10k^ embryos, which are similar to *Hes7* null mutants.

### Transcriptional activities of cyclic genes up-regulated by reduced amount of Hes7 protein in *Hes7*^10k/10k^ embryos

Because Hes7 cyclically represses its own transcription and transcription of cyclic genes including *Lfng*, thereby generating synchronized gene oscillation in PSM^7^, we assumed the transcriptional activities of *Hes7* and *Lfng* would be enhanced in knock-in mice. Thus, we first examined the transcriptional activity of the *Hes7*^10k^ allele. The transcriptional activity can be evaluated by detecting the intron fragments in transcripts^22^,^23^ because the intron sequences in transcripts are spliced out immediately after transcription and the intron fragments are degraded immediately after splicing. We performed *in situ* hybridization with a probe derived from the first intron of *Hes7* to detect regions active in the transcription of *Hes7*. The regions active in the transcription of *Hes7* displayed various patterns in the PSM of wild-type embryos, which is consistent with the expression pattern of *Hes7* mRNA (Fig. 4e). However, the entire PSM of *Hes7*^10k/10k^ embryos was active in the transcription of the *Hes7*^10k^ allele (Fig. 4f). We carried out a quantitative reverse transcription-polymerase chain reaction (RT-PCR) to quantitatively measure transcriptional activity. We detected the sequence of the first intron of *Hes7* in the transcripts, and found that the transcriptional activity of the *Hes7*^10k^ allele in the PSM of *Hes7*^10k/10k^ embryos was substantially up-regulated in comparison with that of the *Hes7* allele in the PSM of wild-type embryos (Fig. 5b). These results suggest that the transcriptional activity of the *Hes7*^10k^ allele is up-regulated in the PSM of *Hes7*^10k/10k^ embryos, probably because of reduced amount of Hes7 protein.

We next examined the expression of *Lfng*, which is one of the target genes of Hes7. As expected, *Lfng* was uniformly expressed throughout the whole PSM of *Hes7*^10k/10k^ embryos (Fig. 4h). We carried out quantitative RT-PCR analyses to quantitatively evaluate *Lfng* expression. We found that the transcriptional activity of *Lfng* was clearly up-regulated in the PSM of *Hes7*^10k/10k^ embryos in comparison with that in the PSM of wild-type embryos (Fig. 5d). The amount of *Lfng* mRNA was also slightly increased in the PSM of *Hes7*^10k/10k^ embryos (Fig. 5c). These results suggest that the reduced amount of Hes7 enhanced the expression of *Lfng*, and that sufficient amounts of *Hes7* are essential for oscillatory gene expression and somite segmentation.

### *Hes7* 3′UTR is essential for somite segmentation clock

**Figure 6:**
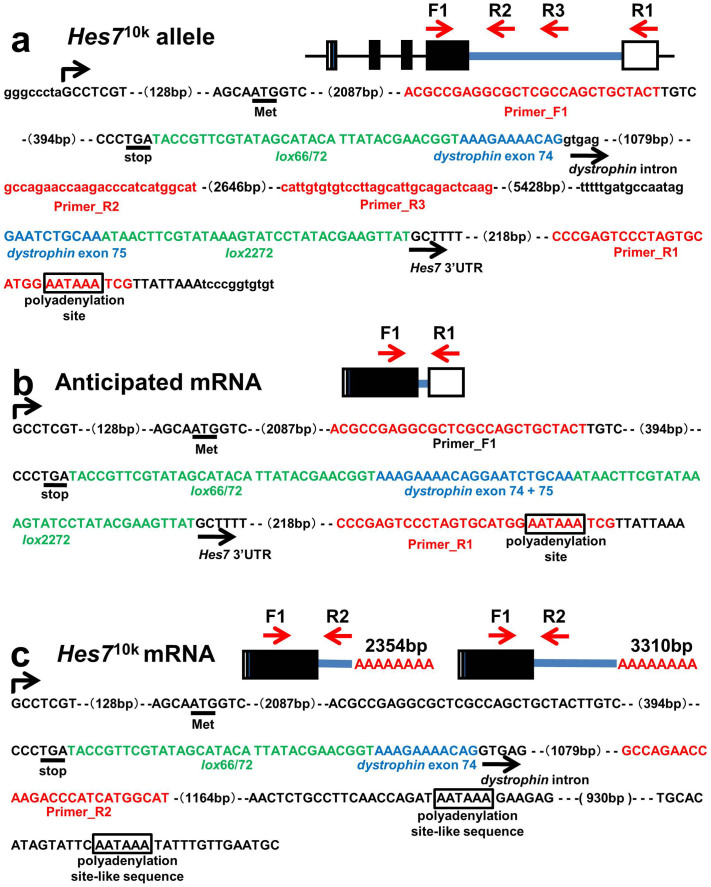
Structure of *Hes7*^10k^ allele and its transcript. (a) Structure of *Hes7*^10k^ allele. The sequence from just upstream of the transcription initiation site of *Hes7* to just downstream of the polyadenylation site of *Hes7* is shown. Upstream and downstream sequences of the *Hes7* gene, and the human *dystrophin* intron sequence are indicated by the small letters. (b) The structure of the anticipated transcripts of the *Hes7*^10k^ allele. (c) The structure of the transcripts of the *Hes7*^10k^ allele that were detected in PSM. The positions of the primers (F1, R1, R2, and R3) are indicated by the red arrows. *Hes7* 5′UTR and 3′UTR are indicated by the open boxes and the *Hes7* coding region is indicated by the closed box. The sequences corresponding to *primers*, mutants of *loxp*, and human *dystrophin* exon are indicated by the red, green, and blue letters. The polyadenylation site and polyadenylation site-like sequences are boxes (AATAAA). The initiation codon (Met) and the termination codon (stop) are underlined.

**Figure 7:**
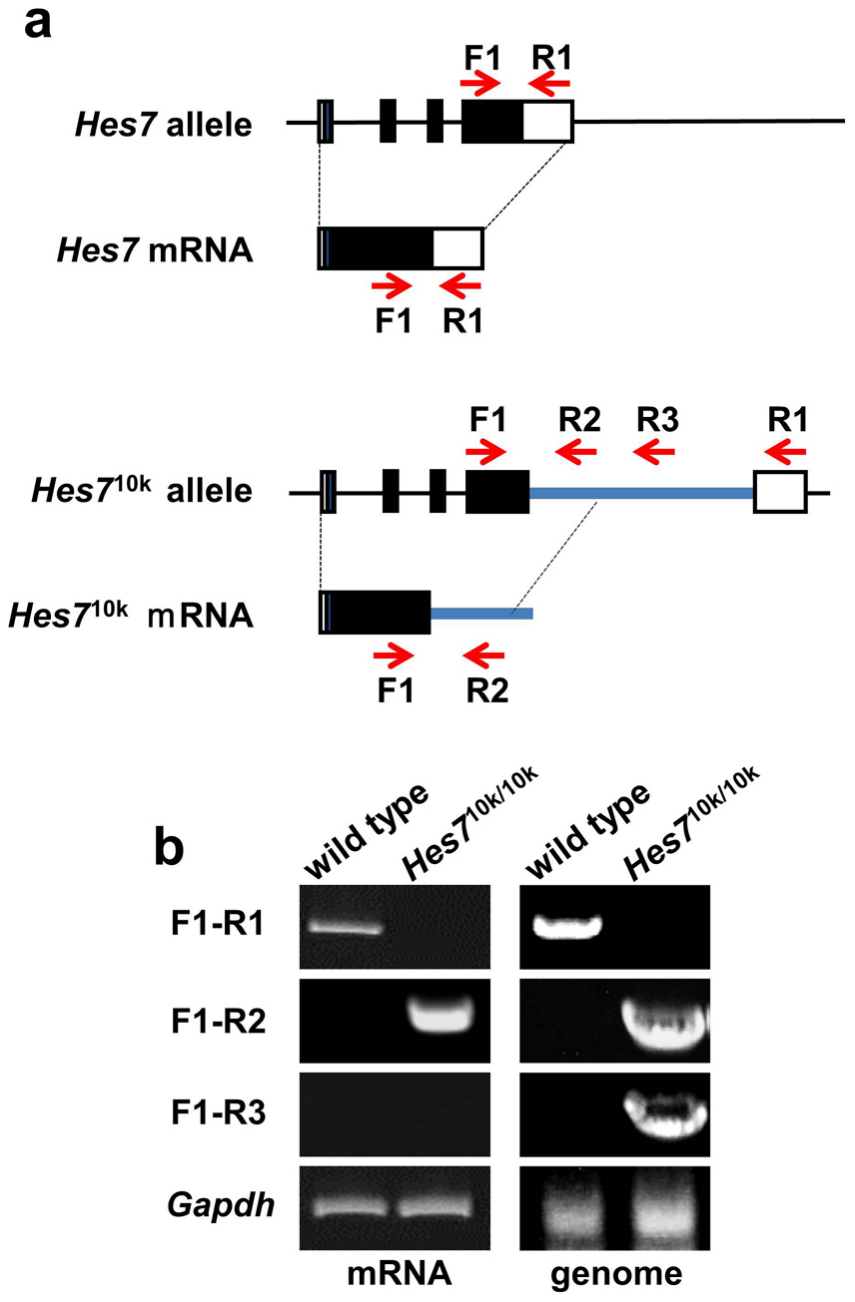
Characterization of transcript of *Hes7*^10k^ allele. (a) Structures of *Hes7* gene and *Hes7* mRNA are provided in upper panel. The bottom panel indicates the structure of the *Hes7*^10k^ allele and its transcript. The intron sequence derived from the human *dystrophin* gene is indicated by the blue lines. The red arrows indicate the position of the primers for RT-PCR analyses. (b) RT-PCR analyses of the transcript of the *Hes7*^10k^ allele. Total RNA was extracted from the PSMs of wild-type or *Hes7*^10k/10k^ embryos, and was analyzed by RT-PCR with the combinations of indicated primers (panels on left). Genomic DNA was extracted from wild-type or *Hes7*^10k/10k^ mice. PCR analyses were carried out with combinations of indicated primers (panels on right). *Gapdh* mRNA was detected as a positive control. The combination of F1 and R1 primers failed to detect the 10 kb band with genome DNA or the mRNA of *Hes7*^10k/10k^ mice.

The 10 kb intron derived from human *dystrophin* in the *Hes7*^10k^ allele was inserted immediately downstream of the stop codon of *Hes7* (Fig. 6a). We carried out RT-PCR with RNAs extracted from the PSMs of wild-type and *Hes7*^10k/10k^ embryos at E 10.5 to check whether this exogenous intron was spliced out to form appropriate mRNAs. We designed a forward primer, F1, in the 4th exon, a reverse primer, R1, in 3′UTR, and two reverse primers, R2 and R3, in the intron derived from *dystrophin* (Fig. 6a–c, 7a). We detected *Hes7* mRNA in wild-type embryos, whereas we failed to obtain a PCR product in *Hes7*^10k/10k^ embryos with the F1 and R1 primers (Fig. 7b). Although a PCR product was detected in *Hes7*^10k/10k^ PSMs by combining the F1 and R2 primers, no products were detected by combining the F1 and R3 primers (Fig. 7b). These results suggest that the human *dystrophin* intron inserted into *Hes7* 3′UTR was not spliced out in *Hes7*^10k/10k^ PSMs, and that the transcripts of the *Hes7*^10k^ allele terminated between the positions of the R2 and R3 primers.

**Figure 8:**
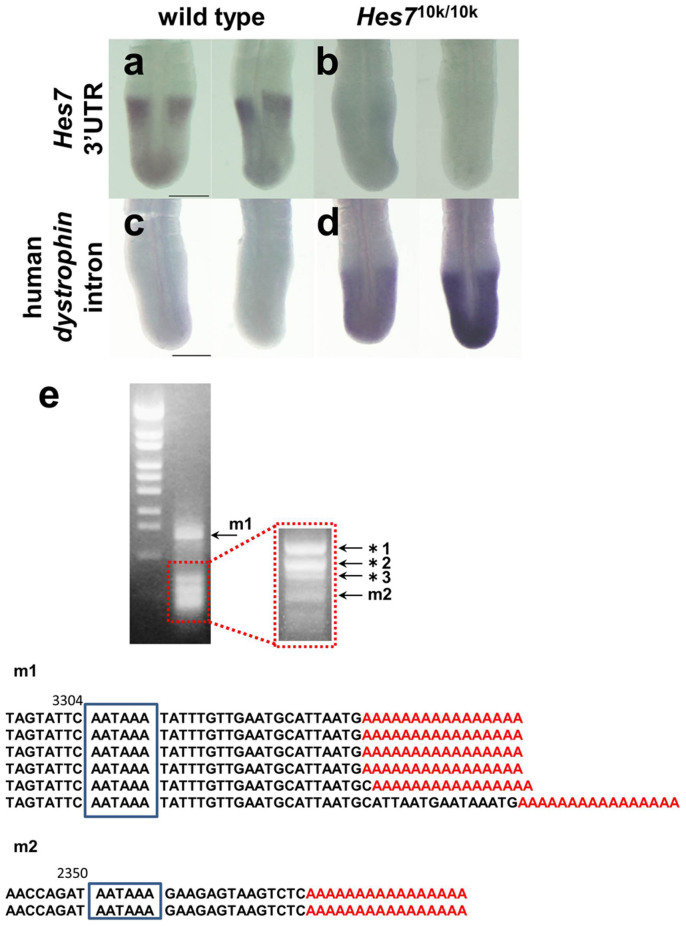
Transcript of *Hes7*^10k^ lacks proper 3′UTR. (a,b) *in situ* hybridization with the probe derived from *Hes7* 3′UTR. *Hes7* mRNA was detected with the 3′UTR probe in the wild-type embryo (*n* = 20) (a), whereas the transcript of *Hes7*^10k^ was not detected with the 3′UTR probe in the *Hes7*^10k/10k^ embryo (*n* = 3) (b). (c,d) *In situ* hybridization with the probe derived from the human *dystrophin* intron. We used a 754 bp fragment of the 5′ part of intron 74 of human *dystrophin*. No signals were detected in the wild-type embryo (*n* = 9) (c). In *Hes7*^10k/10k^ embryo, the transcript of *Hes7*^10k^ was detected in PSM (*n* = 2) (d). Scale bars: 100 μm. (e) 3′RACE analysis of *Hes7*^10k^ transcripts. Total RNA was extracted from the PSM of *Hes7*^10k/10k^ embryos. The 3′ end of the transcript of the *Hes7*^10k^ allele was identified by 3′RACE analysis. Two major bands (m1 and m2), which are indicated by the arrows in the upper panel, contained the sequences in the lower panel. Poly-A tails are indicated by the red As. The other bands denoted by the asterisks contained sequences that were not involved in *Hes7*: *1, family with sequence similarity 69 member B (Fam69b) (650 bp): *2, histone cluster 1, H2ag (His1h2ag) (500 bp): *3, hemoglobin alpha, adult chain 1 (Hba-a1) (400 bp).

We carried out *in situ* hybridization with a probe derived from *Hes7* 3′UTR to further examine the structure of *Hes7* mRNA in *Hes7*^10k/10k^ embryos. We detected *Hes7* mRNA in wild-type embryos, whereas we failed to detect *Hes7* mRNA in *Hes7*^10k/10k^ embryos (Fig. 8a,b). In contrast, we uniformly detected mRNAs in the PSM of *Hes7*^10k/10k^ embryos by using a probe derived from the 5′ end of the *dystrophin* intron (Fig. 8d), and the pattern was similar to the pattern obtained with the probe derived from the coding of *Hes7* (Fig. 4b). No signal was detected with the *dystrophin*-intron probe in wild-type embryos because this intron was adopted by the human *dystrophin* (Fig. 8c). These results support our interpretation that the exogenous intron was not spliced out in the *Hes7*^10k^ transcripts, which were prematurely terminated between the positions of the R2 and R3 primers.

We carried out 3′-rapid amplification of cDNA ends (3′-RACE) to identify the positions of the 3′ end of the transcripts of *Hes7*^10k^. We detected several PCR products, and two of them were derived from the *Hes7* gene (Fig. 8e). One was terminated at a site 2.4 kb downstream of the stop codon. The other was terminated at a site 3.3 kb downstream of the stop codon. Because the 3′ ends of both products were located between the positions of the R2 and R3 primers, this was consistent with the results obtained from RT-PCR analysis (Fig. 6a,b). We found polyadenylation site-like sequences (AATAAA) just upstream of the 3′ ends of both transcripts (Fig. 6c,8e). Thus, these results suggest that sequences similar to the polyadenylation site in the exogenous human *dystrophin* intron were misidentified, thereby leading to premature termination and adding polyadenylation tails to the *Hes7*^10k^ transcripts. *Hes7* 3′UTR was essential to generate sufficient amounts of Hes7 protein to maintain the somite segmentation clock, taking all results into account.

## Discussion

We manipulated the mouse *Hes7* gene in ES cells, with which we generated lines of knock-in mice. The proper 3′UTR of *Hes7* mRNA was replaced by an exogenous sequence derived from the human *dystrophin* intron by modifying the *Hes7* gene. This alteration of 3′UTR reduced the amount of *Hes7* mRNA and Hes7 protein, leading to gene oscillation in PSM being damped and to the failure of periodic somite formation. We therefore concluded that Hes7 played the major role in generating gene oscillation in PSM, where *Hes7* 3′UTR was essential to generate sufficient amounts of Hes7 protein.

We originally attempted to increase the time for mRNA transcription by increasing the gene size of *Hes7*, thereby expanding the oscillation period. We expected that the extended oscillation period would decrease the number of somites and vertebrae and/or increase the size of somites. However, the knock-in mice did not exhibit the expected phenotypes because the inserted introns decreased the amount of *Hes7* mRNA and Hes7 protein in PSM, thereby leading to damped oscillation.

Stauber et al. recently independently attempted to elongate time delay in the segmentation clock^14^. They generated knock-in mice, where the size of the third intron of *Hes7* was increased. However, this modification caused incorrect splicing, thereby drastically reducing the activity of Hes7. Consequently, the homozygous knock-in mutant embryos demonstrated similar phenotypes in somites and axial skeletons as the *Hes7* knock out embryos. Thus, they failed to increase the time delay in knock-in mice. Hanisch et al., tried to rescue the mutant of *her1*/*her7* in zebrafish, which were the orthlogs of *Hes7*, by introducing a *her1* transgene that inserted a sequence derived from human *dystrophin* into its second intron^15^. Unfortunately, they failed to rescue the mutants with the transgene because the modified intron was not correctly spliced out. Nevertheless, they measured the velocity of RNA polymerase II on the allele by using the transgene, and demonstrated that it was 4.8 kb/min, which was much faster than they expected. According to the results, they discussed that the contribution of time taken for transcription to the time delay in the segmentation clock was much shorter than that of time taken for other steps including the splicing and export of mRNA. Hoyle and Ish-Horowicz measured the time to splice and export the mRNA of cyclic genes in mouse, chick and zebrafish PSM^24^. Although they tried to estimate the time to transcribe the genes in vivo, they failed because the velocity of transcription was too fast. The time delay in the feedback loop of Hes7 contains the time for several steps including transcription, mRNA processing, protein translation, protein modification, and mRNA and protein trafficking. These reports concluded that the contribution of time in these steps taken for transcription to the time delay was much smaller than that of time taken for other steps^15^,^24^. However, it is quantitatively difficult to alter the time for these steps because their kinetics is not well understood. Thus, although three groups, including us, failed practically to elongate the time to transcribe *Hes7*^14^,^15^, manipulating the gene size remains a possibility to increase the oscillation period.

Conversely, Takashima et al. tried to shorten time delay by removing the introns from the *Hes7* gene^25^. They estimated that introns led to about a 19-min delay in the *Hes7* gene. The *Hes7* gene has a total length of about 2.8 kb^6^, and RNA polymerase was speculated to have a velocity of 1.1–4.8 kb/min^15^,^16^,^17^,^18^,^19^,^20^. Thus, the time to transcribe introns should be less than 2 min. Most of the time delay created by introns is probably due to the process of splicing. They found that gene oscillation in mutant mice was damped, and that segmentation was severely affected. Thus, they concluded that the time delay derived from introns was essential for gene oscillation in the segmentation clock^25^. The same group also found that deleting two introns from *Hes7*, which contains three introns, reduced the time delay by 5 min, and that the deletion increased the number of somites and vertebrae in the rostral part, whereas somite segmentation was affected in the caudal part^26^. They also confirmed a shorter period of gene oscillation and somite segmentation in vivo. Their results are consistent with predictions by the mathematical model in which severe reduction in time delay abolishes gene oscillation, whereas mild reduction in time delay leads to more rapid but dampened oscillation^25^,^26^. Thus, it is likely that the oscillation period depends on the time delay that is generated by splicing, and that it is the mechanism that controls the oscillation period. However, the possibility that splicing or introns *per se* are essential for a proper oscillation period cannot be denied. Thus, increasing the delay time is essential to clarify the mechanism that controls the period of the segmentation clock.

We could not clarify why the exogenous intron was not properly spliced out and why it prematurely terminated. A possible reason is that the exogenous intron derived from human *dystrophin* is too long to be spliced out. However, this is not likely because this intron is properly spliced out in the *dystrophin* gene in humans^17^, and because the shorter intron (5 kb) also failed to be spliced out. Using other introns instead of those derived from human *dystrophin* may solve the problem.

Nonsense-mediated mRNA decay (NMD) is a well-studied mechanism that selectively eliminates abnormal mRNAs^27^. An intron located downstream of the stop codon is detected in the NMD mechanism, thereby the transcript is rapidly degraded. However, if the distance between the stop codon and the intron is less than 50–55 bp, the transcripts are not degraded by NMD^28^. Therefore, we placed the exogenous intron just downstream of the stop codon of *Hes7* to avoid NMD. The transcripts were not subjected to NMD because the exogenous intron in the mutant *Hes7* transcript was not spliced out in the *Hes7*^10k/10k^ embryo. Thus, the small reduction of *Hes7* mRNA in the *Hes7*^10k/10k^ embryo is not likely due to NMD.

The amount of Hes7 protein in the PSM of *Hes7*^10k/10k^ embryo was reduced, and this was probably responsible for the phenotype in the mutant embryos. Thus, we concluded that *Hes7* 3′UTR is essential for maintaining the amount of Hes7 protein. We recently demonstrated that the 3′UTRs of *Hes7* and *Lfng* were responsible for the differential distribution patterns of their mRNAs in PSM^29^. This is consistent with our findings in this report, which has accumulated evidence on the importance of 3′UTR in gene oscillation. We demonstrated in this research that the amount of *Hes7* mRNA was reduced by 30% in the PSM of *Hes7*^10k/10k^ embryos, whereas the transcriptional activity of *Hes7* was slightly up-regulated (Fig. 4). This suggests that *Hes7* 3′UTR maintains the proper stability of *Hes7* mRNA. It is not likely that the rate at which Hes7 is degraded is increased in *Hes7*^10k/10k^ embryo because the primary structure of Hes7 protein is not altered by mutation in the *Hes7* gene. Thus, the efficacy of export from the nucleus to the cytoplasm or translation of *Hes7* mRNA may be reduced by abnormal 3′UTR. Further studies are needed to clarify the role of *Hes7* 3′UTR.

## Methods

### Generation of *Hes7*^10k/10k^ knock-in mice

We used mutated *loxp* sequences of *lox*71, *lox*2272, and *lox*66. *lox*71 and *lox*2272 are exclusively recombined with *lox*66 and *lox*2272, respectively, in the presence of Cre recombinase^30^,^31^. The targeting vector was constructed by inserting *lox*71, PGK-*neo*, PGK-*tk*, and *lox*2272 just after the stop codon of *Hes7*, and it was transfected to embryonic stem (ES) cells (TT2)^32^. *Hes7*^neo/+^ clones were selected by using 250 μg/ml G418 (Nacalai tesque, Japan), and their genotype were checked with Southern blotting. We placed *lox*66 at the 5′ end and *lox*2272 at the 3′ end of the fragments of the human *dystrophin* gene in the intron vector. We took 11 bp of the 3′ end of exon 74, the consequent intron 74 (20 kb), and 10 bp of the 5′ end of the exon 75 of the human *dystrophin* gene^33^. Vectors for the 10-kb and 5-kb introns were generated by deleting the middle part of the human *dystrophin* intron. The intron vectors were transfected into the *Hes7*^neo/+^ clone with the cre expression vector, and the *Hes7*^20k/+^, *Hes7*^10k/+^, and *Hes7*^5k/+^ clones were selected by using 0.2 μM of FIAU (Wako, Japan). The genotypes were checked with Southern blotting and PCR. We confirmed *Hes7* coding, introns, and the junction of the *Hes7* gene and the exogenous fragment by sequencing. We inserted the *Hes7*^20k/+^, *Hes7*^10k/+^, and *Hes7*^5k/+^ ES cells into the blastocysts of CD1 mice, and generated chimeric mice, which were crossed with CD1 mice to generate *Hes7*^20k/+^, *Hes7*^10k/+^, and *Hes7*^5k/+^ mice. *Hes7*^20k/20k^, *Hes7*^10k/10k^, and *Hes7*^5k/5k^ mice were generated by intercrossing. Genotyping was checked with the following primers of Hes7_F GAGCAATGGTCACCCGGGAGCG, Hes7_R TCTGTAAGGCGGTGGCGGTGGC, and Dys-intron_R GAGCAATGGTCACCCGGGAGCG (Fig. 1b).

Our experiments were approved by the Animal Care Committee of Nara Institute of Science and Technology and conducted in accordance with guidelines that were established by the Science Council of Japan.

### Whole-mount immunostaining and whole-mount *in situ* hybridization

Embryos at E 10.5 were fixed with 4% paraformaldehyde for 3 h for whole-mount immunostaining and they were treated with 1% Triton-X100 (Sigma) and 0.1% H_2_O_2_ for 30 min. We incubated the embryos with guinea pig antibodies against Hes7 (diluted 1:200) for 12 h at 4°C and then with peroxidase-conjugated antibodies against guinea pig IgG for 12 h at 4°C. We visualized the peroxidase activity by using TSA kit#2 (alexa fluor488). Hoechst33258 was used for nuclear staining, and fluorescent images were collected with LSM710 (Carl Zeiss). Whole-mount *in situ* hybridization was carried out as previously described^5^. *Hes7* nascent transcripts and *Hes7*^10k^ nascent transcripts were detected with a probe that corresponds to the first intron of *Hes7*. Mature transcripts of *Hes7* were detected with probes that correspond to the coding or 3′UTR of *Hes7*. *Hes7*^10k^ mature transcripts were detected with probes corresponds to the 5′ part of intron 74 of human *dystrophin*. The images were collected with a digital microscope VHX-5000 (Keyence) (Fig. 3) or a stereoscopic microscope SZX16 (Olympus) (Fig. 4,8).

### RNA extraction

We extracted total RNA from the PSMs of embryos by using a Nucleo Spin RNA XS kit (Macherey-Nagel). cDNAs were synthesized from total RNAs by using SuperScriptII Reverse Transcriptase (Invitrogen) with random primers according to the manufacturer's instructions.

### Quantitative PCR

Quantitative PCR was carried out in the presence of a KAPA SYBR FAST Universal 2X qPCR Master Mix (Nippon Genetics) on a Light Cycler 480 (Roche) under conditions of 95°C for 3 min, and 40 cycles at 95°C for 10 sec, 60°C for 20 sec, and 72°C for 1 sec. *Hes7* mRNA was detected with the primers Hes7 Exon3_F and Hes7 Exon4_R. *Hes7* nascent transcripts were detected with the primers Hes7 Intron1_F and Hes7 Intron1_R. *Lfng* mRNA was detected with the primers Lfng mRNA_F and Lfng_Rv. Lfng nascent transcripts were detected with the primers Lfng intron_F and Lfng_Rv. GAPDH mRNA was quantified with the primers Gapdh_Fw and Gapdh_Rv. The sequences for the primers were: Hes7 Exon3_F CGAAGCTGGAGAAAGCGGAGATACTGGA, Hes7 Exon4_R CCGGACAAGTAGCAGCTGGCGAG, Hes7 Intron1_FW GAGAGGTGGGAAGGGAGG, Hes7 Intron1_RV CTCTGACCCTGCCCTCTTTATACTT, Lfng mRNA_Fw AAGCTCACAGGCAATGTGGT, Lfng_Rv CCGGAGGTTGACGTAGTTGT, Lfng intron_Fw TCCTACCTTTCCCTCTGTGC and Lfng_Rv CCGGAGGTTGACGTAGTTGT.

### RT-PCR analysis

Total RNAs were extracted from individual wild-type, *Hes7*^10k/10^, and *Hes7*^-/-^ embryos at E 10.5. RT-PCR was carried out with LA taq (TaKaRa) under conditions of 94°C for 5 min, and 32 cycles at 94°C for 30 sec, 60°C for 30 sec, and 72°C for 10 min. The sequences of the primers were: Primer_F1 AACCTCCGGAACCCGAAGCTGGAGAAA, Primer_R1 TCAGGGCCAAGGTCTCCAAAACGC, Primer_R2 ATGCCATGATGGGTCTTG GTTCTGGC, and Primer_R3 CGCGACTGGTATGTTCAGATA AGTAAGCAGC, Gapdh_Fw TTCACCACCATGGAGAAGGC, and Gapdh_Rv TTGTCATGGATGACCTTGGC.

### 3′-rapid amplification of cDNA ends (3′RACE)

Total RNAs were extracted from individual *Hes7*^-/-^ embryos at E 10.5. cDNAs were synthesized from total RNAs by using SuperScriptII Reverse Transcriptase (Invitrogen) with Oligo dT-3sites Adaptor Primer (Invitrogen) according to the manufacturer's instructions. The 3′ fragments were amplified with KOD plus (Toyobo) under conditions of 95°C for 5 min, and 32 cycles at 95°C for 30 sec, 60°C for 30 sec, and 72°C for 5 min. We used 1 μl of solution of the RT reaction as a template for the first amplification. We used 1 μl of solution of the first reaction as a template for the first nested reaction, and used 0.5 μM of each primer (3′RACE_R1/Adaptor). A second nested reaction was carried out using 1 μl of solution of the first nested reaction (1:100 dilution) with 0.5 μM of each primer (3′RACE_R2/Adaptor) under the same conditions to ensure specificity. The sequences of the primers were: Oligo dT-3sites Adaptor Primer GGCCACGCGTCGACTAGTACT TTTTTTTTTTTTTTTT, Adaptor GGCCACGCGTCGACTAGTAC, 3′RACE_R1 GATCATTTGTCAGTGAAGACCATGGTAGAG, and 3′RACE_R2 TGAGCTCATTGGT CAAGAGCCCTG.

### Skeletal preparation

The cartilages of newborn mice were stained with alcian blue and their bones were stained with alizarin red, after being fixed in 95% ethanol^5^.

## Author Contributions

T.F., T.M. and Y.N. performed most of the experiments. T.M. and Y.S. performed the mathematical analyses in supplementary note and the statistical analyses. M.S. and K.K. generated knock-in mice. T.M., Y.N. and Y.B. conceived and designed the experiments. Y.B. wrote the manuscript. All authors reviewed the manuscript.

## Supplementary Material

Supplementary Information Supplementary Note
