# Supplementary material for: Hes7 3′UTR is required for somite segmentation function
Source: Sci Rep. 2014 Sep 24;4:6462. doi: 10.1038/srep06462 (PMC4173035; doi:10.1038/srep06462)
Supplement: Supplementary Information — Supplementary Note [file srep06462-s1.doc]

Supplementary information for

***Hes7* 3’UTR is required for somite segmentation function**

Takeshi Fujimuro, Takaaki Matsui, Yasuhide Nitanda, Tatsuro Matta, Yuichi Sakumura, Michiko Saito, Kenji Kohno, Yasukazu Nakahata, and Yasumasa Bessho

# Mathematical model

We computed a simple mathematical equation (Lewis, 2003) to examine the extent of dependence of the period of *Hes7* oscillatory expression on transcription delay. The amount of *Hes7* protein at time
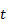
,
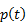
, increases in proportion to the amount of *Hes7* mRNA,
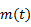
, and conversely, *Hes7* mRNA is suppressed by *Hes7* protein. By taking into consideration degradation and time delays in protein and mRNA syntheses, protein and mRNA obey ordinary differential equations:

|  | 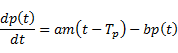 |  |
| --- | --- | --- |
|  | 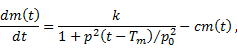 |  |

where
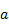
 represents the protein production rate per mRNA molecule,
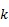
 represents the maximum rate of mRNA, and
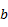
 and
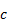
 are degradation rates. Here,
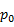
 is a gain parameter,
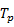
 is the time delay for protein generation, and
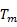
 is that for mRNA generation. The parameter values used in the simulation are listed in Table S1.

The delay parameter,
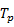
, includes the elapsed time of transcription. We computed the oscillation period of the *Hes7* expression with various values of
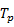
 (Figure S1) with the mathematical model. As the transcription rate was estimated at 1.1-4.8 kb/min, the delay increased by about 9-2.1 min, respectively, when the 10 kb intron was inserted into the wild type. The model revealed that the 9 min of additional delay time extended the period of *Hes7* expression by 20.2 min, whereas the 2.1 min of additional delay time extended the period by 4.9 min. This suggests that the incremental time in the period takes about twice the additional delay time, which is consistent with the findings in a previous report (Lewis, 2003).

**Figure S1.**　Dependence of period of *Hes7* oscillatory expression on transcription delay. The vertical axis represents the averaged period computed with the mathematical model and the horizontal axis is the additional delay time of *Hes7* mRNA transcription. The model indicates a 114.1 min period for the wild type (delay = 0) while it predicts a 134.3 min or 119.0 min period with 9 min or 2.1 min of additional delay time in transcription, respectively.

**Table S1. Representative simulation parameter values.**

| **Parameter** | **Value (unit)** | **Meaning** | **Reference** |
| --- | --- | --- | --- |
| 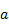 | 0.5 (molecules/min) | Production rate for protein | Kim et al. 2011 |
| 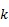 | 33 (molecules/min) | Production rate for mRNA | Hirata et al. 2004 |
| 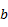 and 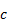 | 0.11 (1/min) | Degradation rate for NICD, mRNA, and protein | Kim et al. 2011 |
| 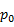 | 13 (min) | Gain value of *Hes7* to inhibit protein production | Kim et al. 2011 |
| 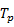 | 15 (min) | Time delay for transcription, nuclear export, and protein production | Kim et al. 2011 |
| 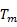 | 20 (min) | Time delay for *Hes7* nuclear translocation | Kim et al. 2011 |

# References

Lewis, J. (2003). Autoinhibition with transcriptional delay: a simple mechanism for the zebrafish somitogenesis oscillator. Curr Biol *13*, 1398-1408.

Hirata, H., Bessho, Y., Kokubu, H., Masamizu, Y., Yamada, S., Lewis, J., Kageyama, R. (2004). Instability of Hes7 protein is crucial for the somite segmentation clock. Nat Genet *36*, 750-754.

Kim, W., Matsui, T., Yamao, M., Ishibashi, M., Tamada, K., Takumi, T., Kohno, K., Oba, S., Ishii, S., Sakumura Y., and Bessho, Y. (2011) The period of the somite segmentation clock is sensitive to Notch activity. Molecular Biology of the Cell, *18*, 3541-3549
